# XIAP Antagonist Embelin Inhibited Proliferation of Cholangiocarcinoma Cells

**DOI:** 10.1371/journal.pone.0090238

**Published:** 2014-03-06

**Authors:** Cody J. Wehrkamp, Ashley R. Gutwein, Sathish Kumar Natarajan, Mary Anne Phillippi, Justin L. Mott

**Affiliations:** Department of Biochemistry and Molecular Biology, Fred and Pamela Buffett Cancer Center, University of Nebraska Medical Center, Omaha, Nebraska, United States of America; Roswell Park Cancer Institute, United States of America

## Abstract

Cholangiocarcinoma cells are dependent on antiapoptotic signaling for survival and resistance to death stimuli. Recent mechanistic studies have revealed that increased cellular expression of the E3 ubiquitin-protein ligase X-linked inhibitor of apoptosis (XIAP) impairs TRAIL- and chemotherapy-induced cytotoxicity, promoting survival of cholangiocarcinoma cells. This study was undertaken to determine if pharmacologic antagonism of XIAP protein was sufficient to sensitize cholangiocarcinoma cells to cell death. We employed malignant cholangiocarcinoma cell lines and used embelin to antagonize XIAP protein. Embelin treatment resulted in decreased XIAP protein levels by 8 hours of treatment with maximal effect at 16 hours in KMCH and Mz-ChA-1 cells. Assessment of nuclear morphology demonstrated a concentration-dependent increase in nuclear staining. Interestingly, embelin induced nuclear morphology changes as a single agent, independent of the addition of TNF-related apoptosis inducing ligand (TRAIL). However, caspase activity assays revealed that increasing embelin concentrations resulted in slight inhibition of caspase activity, not activation. In addition, the use of a pan-caspase inhibitor did not prevent nuclear morphology changes. Finally, embelin treatment of cholangiocarcinoma cells did not induce DNA fragmentation or PARP cleavage. Apoptosis does not appear to contribute to the effects of embelin on cholangiocarcinoma cells. Instead, embelin caused inhibition of cell proliferation and cell cycle analysis indicated that embelin increased the number of cells in S and G2/M phase. Our results demonstrate that embelin decreased proliferation in cholangiocarcinoma cell lines. Embelin treatment resulted in decreased XIAP protein expression, but did not induce or enhance apoptosis. Thus, in cholangiocarcinoma cells the mechanism of action of embelin may not be dependent on apoptosis.

## Introduction

Cholangiocarcinoma is a liver tumor with cellular features of bile duct epithelial cells and is the second most common primary liver cancer. Biliary tract inflammation predisposes to cholangiocarcinoma, although most patients do not have recognized underlying liver disease at the time of diagnosis. Chemotherapy has been shown to prolong survival, but only modestly [1], and five-year survival remains less than 10%. This may be due to decreased tumor cell death in response to chemotherapy. A number of mechanisms contribute to apoptosis resistance, including overexpression of the caspase-inhibitory protein X-linked inhibitor of apoptosis protein (XIAP).

XIAP is an E3 ubiquitin-protein ligase that binds and inhibits caspases 3, 7, and 9 [2], [3]. XIAP is ubiquitously expressed at the mRNA level [4] and has been shown to be induced in cholangiocarcinoma cells by the inflammatory mediator IL-6 [5]. XIAP protects cholangiocarcinoma cells from apoptosis induced by chemotherapeutic drugs [5] and by the death receptor ligand TNF-related apoptosis-inducing ligand (TRAIL) [6]. Treatment of cholangiocarcinoma cells with the small molecule triptolide resulted in decreased XIAP protein levels and increased sensitivity to TRAIL [7]. Together, these data suggest that targeting XIAP in cholangiocarcinoma cells increases sensitivity to apoptosis. XIAP's antiapoptotic effects are overcome upon mitochondrial membrane permeabilization and release of SMAC/DIABLO [8], a protein that binds the BIR3 domain of XIAP [9], [10].

The small molecule embelin has been found to inhibit XIAP and computer modeling as well as fluorescence polarization competition assays suggest it binds the SMAC-binding pocket of XIAP [11]. Treatment with embelin has been shown to sensitize cells to apoptosis through TRAIL, chemotherapy, and targeted therapy plus cFLIP knockdown. Further, embelin treatments decreased XIAP protein levels in leukemia cells [12]. Based on these findings, embelin has been described as an XIAP antagonist. However, alternate/additional mechanisms of embelin action have been described, including inhibition of NF-kB [13] and inhibition of Akt/mTOR/S6K1 [14].

In this study, we sought to assess the effects of embelin on XIAP protein levels, apoptosis, and proliferation in cholangiocarcinoma cells. While embelin decreased cellular XIAP protein levels, caspase activity was not increased. Proliferation was inhibited by embelin and cells were arrested in S and G2/M phases. These observations indicate that embelin reduced tumor cell survival and proliferation, but did not increase apoptosis.

## Results

**Figure 1:**
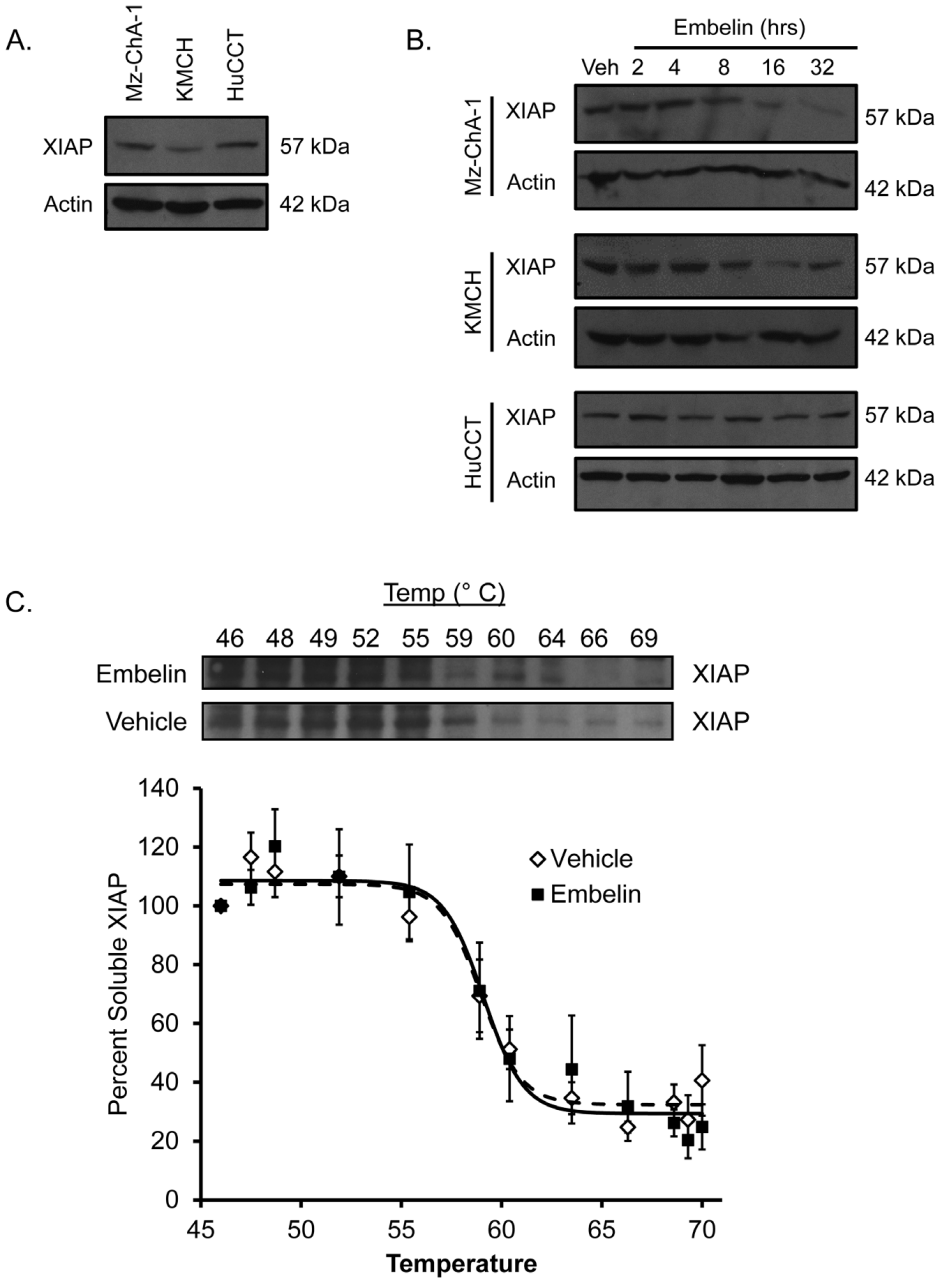
Embelin caused XIAP degradation in cholangiocarcinoma cell lines. (A) Immunoblot of XIAP in untreated cholangiocarcinoma cell lines. Actin was included as a loading control. Apparent molecular weight for each band is indicated to the right. (B) Cells were treated with 15 µM embelin in DMSO or DMSO alone (Veh) for the indicated times. Whole cell lysates were blotted for XIAP and actin. (C) For the cellular thermal shift assay, Mz-ChA-1 cells were lysed by freeze-thaw and then incubated with embelin (50 µM) or DMSO (Vehicle) for 30 minutes and separated into 20 µL aliquots. Aliquots were heated to the indicated temperatures and cooled to room temperature and soluble XIAP measured by immunoblot. Band intensity was determined by densitometry of scanned films and data are plotted compared to the signal intensity observed at 45°C (100%). Data are fitted using the Boltzman function; the dashed line indicates the fit for vehicle-treated samples, the solid line for embelin-treated samples. Blot is representative of four replicates used in the graph.

To assess the potential for antagonism of XIAP in cholangiocarcinoma cells, we first determined XIAP expression at the protein level in several cell lines. XIAP protein was expressed in all three cell lines with highest expression in Mz-ChA-1 cells and HuCCT cells, and somewhat lower XIAP protein levels in KMCH cells (Fig. 1A). Upon treatment with embelin, cellular XIAP protein levels decreased with time in Mz-ChA-1 and KMCH cells, while XIAP was essentially unchanged in HuCCT cells treated with embelin for up to 32 hours (Fig. 1B).

We sought evidence that embelin binds directly to XIAP protein in our cells by employing the cellular thermal shift assay [15]. This assay is based on the observation that ligand binding often stabilizes the cognate target protein [16]–[19]. The cellular thermal shift assay measures heat-induced protein denaturation in the absence and presence of the small molecule ligand. In this case, lysed Mz-ChA-1 cells were incubated with vehicle or embelin and XIAP denaturation was measured by loss of solubility upon heat treatment. We observed that XIAP protein in cell lysates became insoluble at about 60°C. The denaturation temperature was not different in the presence or absence of embelin (61.0+/−1.4 °C versus 59.9+/−0.7°C, respectively; p = 0.49 by *t*-test; Fig. 1C).

**Figure 2:**
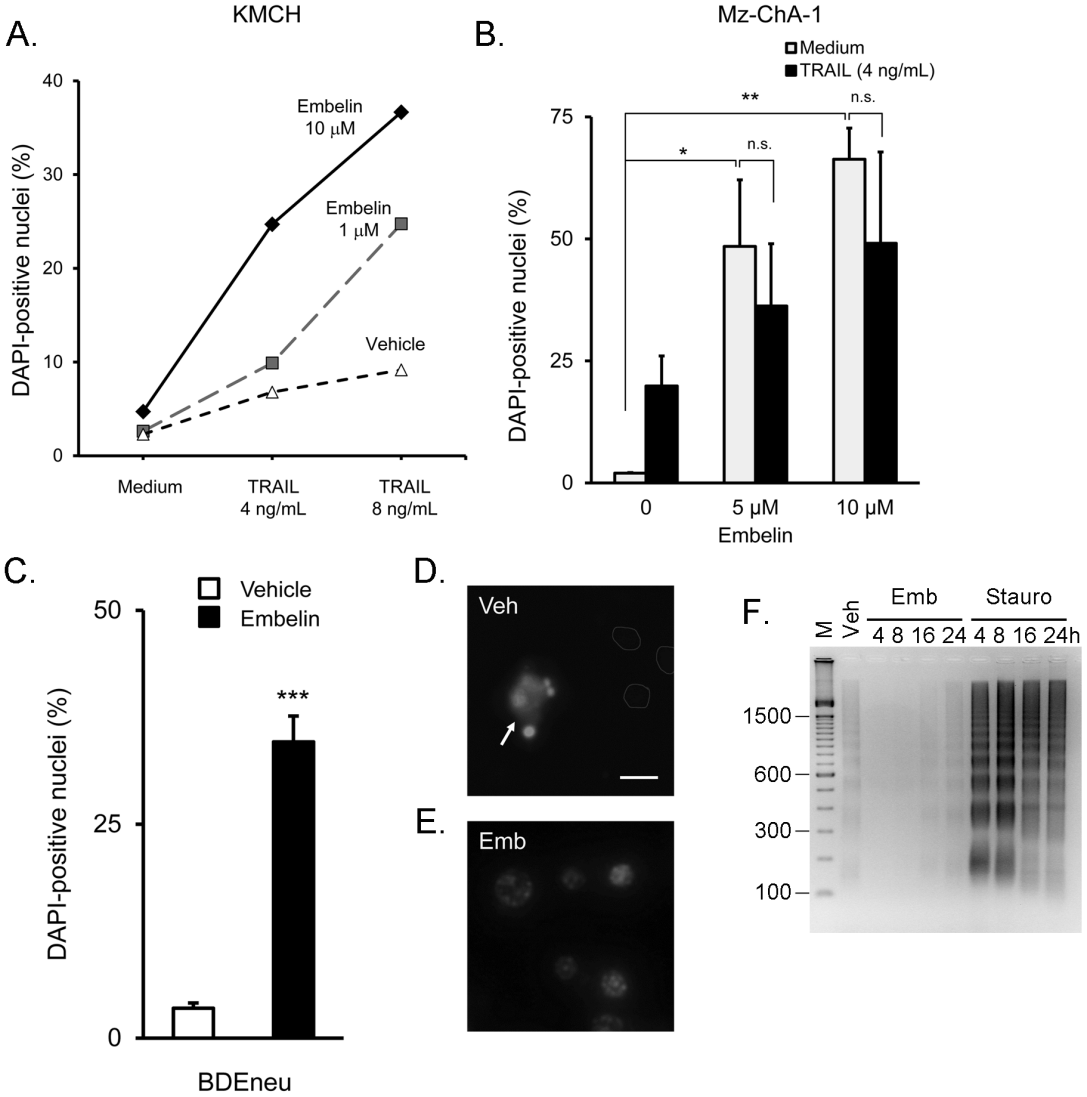
Embelin induced altered nuclear morphology in cholangiocarcinoma cell lines. (A) KMCH cells were treated for 24 hours with TRAIL at the indicated concentrations with or without embelin (1 and 10 µM). Cells were then stained with DAPI and bright nuclei were counted as a percentage of total nuclei. Data from one experiment are plotted as percent DAPI-positive nuclei on the vertical axis. (B) Mz-ChA-1 cells were treated with TRAIL (4 ng/mL) or medium for 24 hours with 5 or 10 µM embelin, and DAPI-positive nuclei counted as a percent of total cells. Data are mean of 3 experiments +/− standard error of the mean. n.s. = not significantly different. * p<0.05, ** p<0.01 by ANOVA with Bonferroni compared to medium alone. (C) Rat BDEneu cholangiocarcinoma cells were treated with DMSO (Vehicle; open bar) or embelin (50 µM, filled bar) for 48 hours, followed by DAPI staining. Data are mean of 3 experiments +/− SEM. *** p<0.001 compared to vehicle, Students *t*-test. (D) Vehicle-treated Mz-ChA-1 cells were stained with DAPI and imaged by epifluorescence without fixation. Healthy nuclei (indicated by grey outlines) did not stain with DAPI while a sporadic apoptotic nucleus (arrow) was brightly stained. Bar = 10 µm. (E) DAPI-positive nuclei of Mz-ChA-1 cells treated with embelin (15 µM for 24 hours) did not show characteristic apoptotic fragmentation or pyknosis. (F) Mz-ChA-1 cells were treated with DMSO (Veh), embelin (15 µM), or staurosporine followed by analysis of DNA fragmentation on a 2% agaraose gel. Vehicle treatmetn was for 24 hours. Embelin and staurosporine treatments were for 4, 8, 16, and 24 hours. M = 100 bp DNA marker. The gel was stained with ethidium and photographed and the image was inverted to show DNA as a dark signal on a light background. Images in Panel D, E, and F were adjusted for brightness and contrast to ensure that features were visible and the entire image was treated equally.

Previous studies have found that siRNA-mediated depletion of XIAP was sufficient to sensitize cholangiocarcinoma cells to apoptosis. We tested cell treatment with embelin or embelin plus TRAIL in KMCH (Fig. 2A) and Mz-ChA-1 cells (Fig. 2B) by quantifying altered nuclear morphology after staining with the DNA-binding dye, 4′-6-diamidino-2-phenylindole (DAPI). The addition of embelin (1–10 µM) increased TRAIL-induced DAPI-positive nuclei in both cell types. Interestingly though, in Mz-ChA-1 cells, embelin alone appeared to have as much effect as embelin plus TRAIL (Fig. 2B). Additional testing of the highly tumorigenic rat-derived BDEneu cell line also showed increased numbers of DAPI-positive nuclei after embelin treatment (Fig. 2C). This suggested embelin may have single-agent activity in cholangiocarcinoma cells. Single-agent activity was somewhat unexpected and (in conjunction with the caspase data, see below) prompted us to closely examine the nuclear staining. Untreated live Mz-ChA-1 cells stained with DAPI showed very low nuclear fluorescence (unstained nuclei outlined), while a sporadic apoptotic nucleus showed bright staining and obvious fragmention (Fig. 2D). Close examination of nuclei in embelin-treated cells revealed DAPI-positive staining with local regions of bright signal, however nuclei did not appear fragmented or condensed, and were not consistent with apoptotic nuclei (Fig. 2E).

Because apoptosis is a process, assessment at a single time point may not accurately capture the apoptotic signal. We have performed a time course of DNA laddering upon embelin treatment (4, 8, 16, and 24 hours) compared to the positive control staurosporine over the same time. The results demonstrate minimal DNA laddering in vehicle (DMSO) treated cells at 24 hours (Veh) that was similar to the laddering seen in embelin-treated cells at 24 hours. In contrast, the kinase inhibitor staurosporine was included as a positive control and showed rapid formation of a DNA ladder with ∼180 bp spacing, consistent with apoptotic internucleosomal fragmentation (Fig. 2F). The results of this experiment support the previous conclusions based on DAPI staining and add additional evidence that the nuclear morphology changes we initially observed were unlikely to reflect apoptosis.

**Figure 3:**
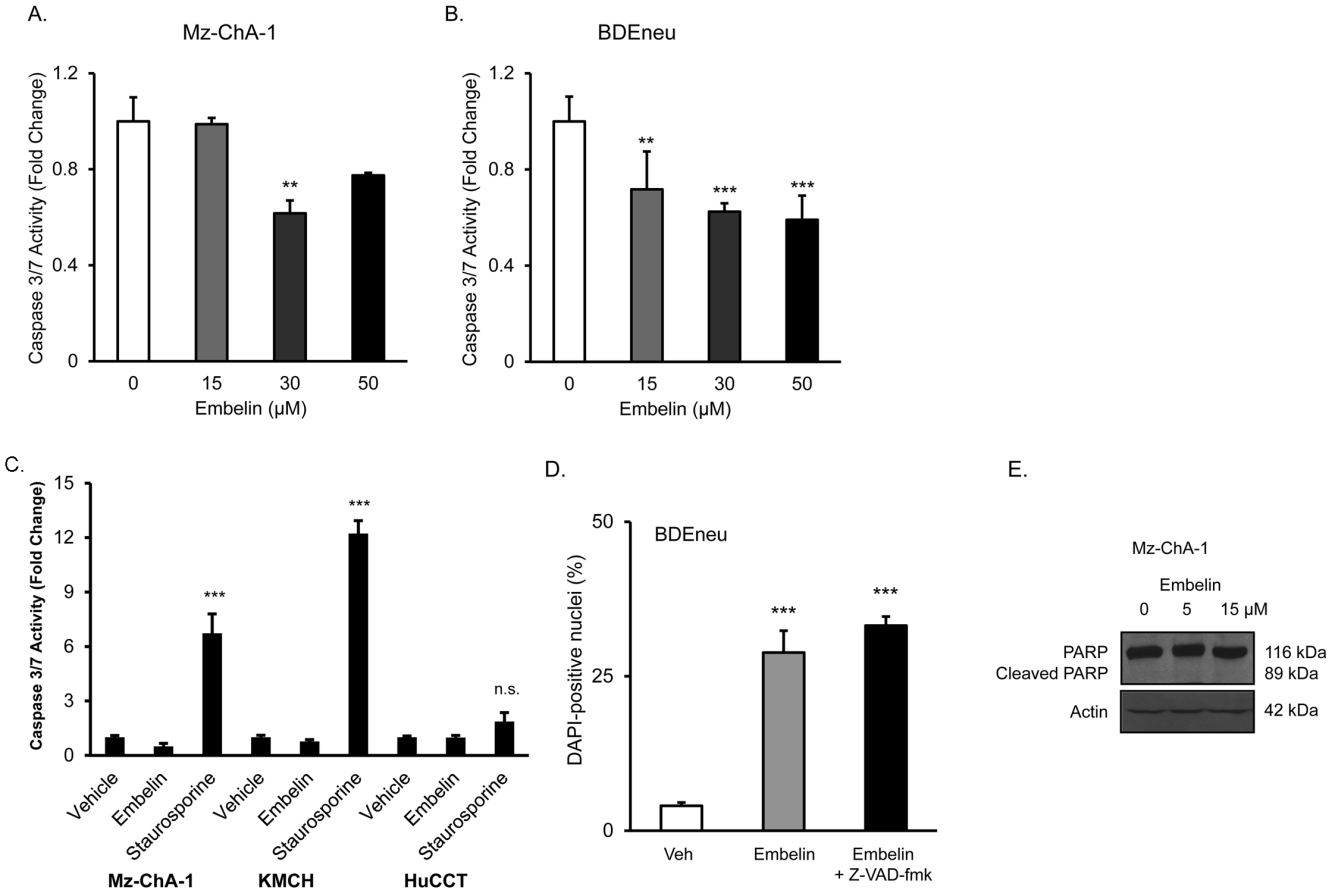
Embelin partially inhibited caspase activation and did not induce caspase-dependent cell death in cholangiocarcinoma cells. (A) Mz-ChA-1 cells were treated with embelin for 24 hours and caspase 3/7 activity measured biochemically. Untreated cells were used for comparison and caspase activity in untreated cells normalized at 1.0. (B) BDEneu cells were treated with embelin (48 hours) and caspase 3/7 activity measured. (C) Caspase 3/7 activity was measured at an earlier time point (4 hours) in Mz-ChA-1, KMCH, and HuCCT cells to test for early caspase activation. Following 4 hours of vehicle, embelin (15 µM) or staurosporine (1 µg/mL), caspase 3/7 activity was measured biochemically. (D) BDEneu cells treated with 50 µM embelin for 48 hours were assayed for DAPI-positive nuclei with and without co-treatment with the caspase inhibitor Z-VAD-fmk (50 µM). DAPI-positive nuclei are presented as percent of total cells, n = 3, mean +/− SEM. Comparison of embelin versus embelin+Z-VAD-fmk was not significantly different. Panels A, B, C & D data are mean of 3 or 4 experiments +/− SEM; ** p<0.01, *** p<0.001 versus vehicle, ANOVA with Bonferroni correction. (E) Mz-ChA-1 cells were treated with embelin (5–15 µM) in DMSO or DMSO alone (Veh) for 24 hours. Whole cell lysates were blotted for PARP. Actin was included as a loading control. Apparent molecular weight for each protein is indicated to the right.

Based on the known function of XIAP in inhibiting caspase activity, it was anticipated that embelin treatment would increase caspase activation and can increase the levels of cleaved poly (ADP-ribose) polymerase (PARP), a marker of caspase-induced apoptosis. Surprisingly, treatment of Mz-ChA-1 cells with embelin did not result in increased caspase 3/7-like hydrolase activity, but instead caused decreased caspase activation at 30 µM (Fig. 3A). This observation was repeated in BDEneu cells, which also showed inhibition rather than activation of caspase 3/7 (Fig. 3B). Caspase actvity was then assessed at an earlier time point, 4 hours, in case caspase activation was an early rather than late event. Embelin treatment did not increase caspase activity at 4 hours, while the positive control staurosporine caused robust caspase activity in Mz-ChA-1 and KMCH cells (Fig. 3C). Staurosporine did not increase caspase activity to a significant degree in HuCCT cells, possibly indicating resistance or slower apoptosis kinetics in HuCCT cells. To determine if embelin-induced nuclear DAPI staining was caspase dependent, we treated BDEneu cells with vehicle, embelin, or embelin plus the pan-caspase inhibitor Z-VAD-fmk and quantified DAPI-positive nuclei. Embelin treatment resulted in nuclear changes in the presence or absence of Z-VAD-fmk (Fig. 3D), consistent with morphology changes that were not caspase-dependent. Control experiments using the same Z-VAD-fmk concentration confirmed that the inhibitor blocked caspase activity (data not shown). Next, we tested whether embelin treatment affected total PARP protein levels or PARP cleavage in Mz-ChA-1 cells. Clearly, there was no change in the levels of PARP or cleaved PARP with embelin treatment (Fig. 3E). Together, these results suggest that embelin treatment did not alter caspase activity.

**Figure 4:**
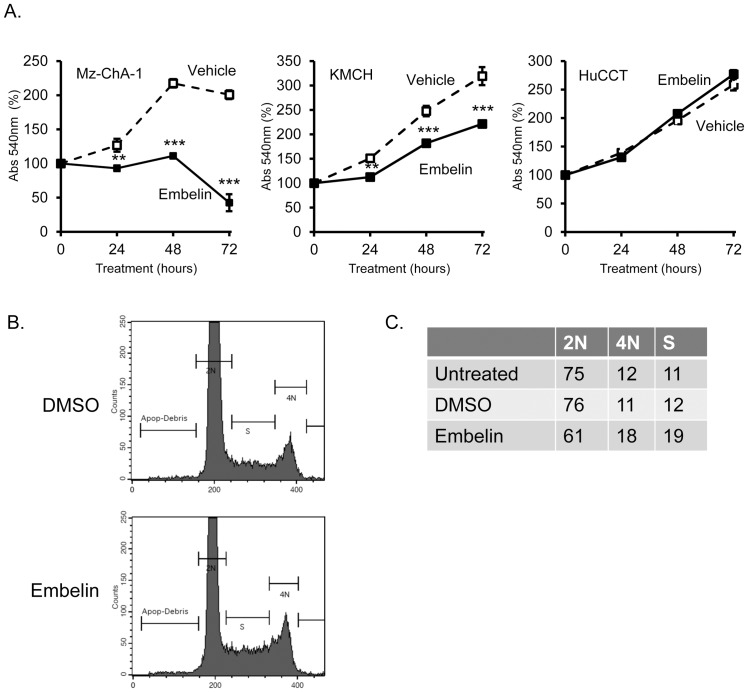
Inhibition of proliferation and cell cycle arrest by embelin. (A) Cell proliferation was measured by MTT and cell number measured by absorbance at 540 nm (Abs 540 nm). Signal represents the mean (n = 4) +/− standard error of the mean, normalized to the starting value (day 0, set at 100%). Cells treated with embelin (15 µM) are plotted with a solid line and filled symbols and vehicle-treated cells are plotted with a dashed line and open symbols. ** p<0.01 and *** p<0.001 versus vehicle at the same time point, ANOVA with Bonferroni correction. Values for HuCCT were not significantly different at any time point. (B) Cell cycle analysis of Mz-ChA-1 cells was performed by propidium iodide staining followed by flow cytometry. A histogram of propidium iodide stained cells is shown for DMSO-treated and embelin-treated cells (15 µM, 24 hours). (C) Quantitation of the percentage of cells with 2N or 4N nuclear DNA content, and cells that are in S phase (DNA content intermediate between 2N and 4N). Representative experiment of 3 independent treatments.

Embelin has been shown to inhibit cell proliferation in cancer cells [20]–[22]. We tested the effect of embelin on Mz-ChA-1 cell growth, using the MTT assay. Growth was significantly reduced, initially apparent as no increase in cell number at 24 or 48 hours, followed by a significant reduction in the number of viable cells at 72 hours in the presence of 15 µM embelin (Figure 4A). Growth inhibition was also apparent in KMCH cells at 24–72 hours, though to a smaller extent than in Mz-ChA-1 cells. HuCCT cells were found to be resistant to the growth-inhibitory effects of embelin, similar to the lack of effect of embelin on XIAP protein content in these cells (see Fig. 1B). To further analyze the effect of embelin on proliferation, investigation of cell cycle progression was performed using propidium iodide staining followed by flow cytometry. Mz-ChA-1 cells were chosen based on their response to embelin treatment in growth assays. In vehicle-treated cells (DMSO), 76% of cells were in the G0/G1 phase (2N), with the remaining cells divided between S phase and G2/M (4N). Treatment with 15 µM embelin caused cell cycle arrest and an increase in the percentage of cells in G2/M as well as an increase in the percentage of cells in S phase. Correspondingly, a decrease in the number of cells in G0/G1 was observed (Fig. 4B and 4C).

## Discussion

The results of this study relate to effects on proliferation of cholangiocarcinoma cells upon embelin treatment. Our results demonstrated that embelin decreased cellular XIAP protein levels, caused a caspase-independent change in nuclear morphology, decreased proliferation, and slowed progression through the cell cycle. Each of these findings will be discussed below.

Embelin has been described to have numerous activities, including antifertility [23] and analgesia [24] functions. Recently, embelin has received attention as an antitumor agent that promotes apoptosis [11], [13], [25], [26] and decreases proliferation [12], [21], [27], [28]. In a computational screen for structures that bind XIAP, embelin was selected for further characterization. Embelin could compete with SMAC for XIAP binding and in prostate tumor cells (PC3) caused loss of cell growth, increased apoptosis (defined as annexin V-positive, propidium iodide-positive cells), and an increased percentage of cells with activated caspase 9 [11]. In a pancreatic cancer cell line, combined treatment with an antisense oligonucleotide to cFLIP, embelin, and TRAIL decreased cell viability compared to cFLIP antisense and TRAIL alone in a tetrazolium-based assay [25]. Because XIAP has a strong effect in cholangiocarcinoma cell lines to protect against cell death, we tested the effect of embelin on XIAP protein levels in human cholangiocarcinoma cell lines and found that embelin caused a reduction in XIAP in Mz-ChA-1 and KMCH cells.

The differential effect of embelin treatment on XIAP protein levels depending on the cell line tested is consistent with literature reports. Embelin treatment of the leukemia cell line HL 60 caused a reduction in XIAP protein levels and increased caspase 3 and caspase 9 cleavage [12]. However, in glioma cell lines, embelin did not significantly alter XIAP protein levels [26]. In a breast cancer cell line overexpressing ErbB2, embelin alone decreased the viability of cells (tetrazolium), although siRNA to XIAP did not. Combined treatment with traztuzumab (an antagonistic ErbB2 antibody) and embelin had no effect while siRNA to XIAP plus traztuzumab increased apoptosis, suggesting that embelin does not simply mimic loss of XIAP [29]. Embelin treatment of PC3 prostate cancer cells did not decrease XIAP protein levels, and did not increase caspase 9 activation (alone or combined with ionizing radiation) although there was an increase in annexin V and propidium iodide double-positive cells [21]. Thus, the effect of embelin on XIAP protein depends on the context. Similarly, the effect on cell viability of embelin alone or in combination treatments varies.

We next sought evidence of a direct interaction of embelin with XIAP in our cells. We utilized the recently-described cellular thermal shift assay [15] to assess the stability of XIAP in the presence or absence of embelin. In our experiments, however, embelin did not reproducibly alter the stability of XIAP. Thus, we were unable to confirm direct binding. This can be interpreted either as a lack of direct binding, or that binding does not significantly stabilize XIAP structurally. In previous heteronuclear single quantum coherence spectroscopy experiments, embelin was found to alter the spectrum of the XIAP BIR3 domain, suggesting a physical interaction [11]. The lack of stabilization in the complex cell lysate (this study) does not rule out a direct interaction, and similarly, observation of a direct binding interaction in a single component system does not answer the question of binding in the cellular environment.

Based on the role of XIAP in preventing cholangiocarcinoma cell apoptosis, we hypothesized that embelin would increase cell death in combination with TRAIL. Initial experiments indeed showed that an increased percentage of cells had altered nuclear morphology upon embelin treatment, measured by the DNA dye DAPI. However, careful analysis confirmed that the altered morphology did not reflect increased apoptotic nuclei. Binding of DAPI to DNA is known to result in increased fluorescent signal over soluble unbound DAPI [30]. Altered nuclear morphology is a hallmark of apoptosis, and can be easily visualized by DAPI staining as increased fragmentation, compaction of the nuclear signal, and increased staining intensity. Indeed, an advantage of using DAPI as a DNA stain in apoptosis measurement is the observation that many viable cells exclude the dye but dying cells take up DAPI and fluoresce brightly, thus providing a strong signal with low background staining of viable nuclei. Notably, some living cells take up DAPI, possibly through the transporters organic cation transporter-1 (OCT1) [31] and multidrug and toxin extrusion proteins (MATE1 and MATE2) [32], and most cells will gradually accumulate DAPI over time. Thus, a brightly stained nucleus is not definitive evidence of apoptosis. Additional morphological features can be used then to distinguish brightly-stained living cells from brightly-stained apoptotic cells, including fragmentation and condensation of the nucleus. Altered nuclear morphology is also observed during different phases of the mitotic or meiotic cell cycle (e.g., see [33] and [34]) and with different chromatin state (heterochromatin versus euchromatin). Thus, an alternate measure of apoptosis is important, such as DNA fragmentation, biochemical assessment of caspase activity, and immunoblot analysis of cleaved PARP levels. Importantly, in our cells, embelin treatment did not induce DNA fragmentation and caused inhibition, not activation of caspases, and did not increase the levels of cleaved PARP. Further, inhibition of caspase activity did not alter embelin-induced nuclear staining. Thus, we interpret the altered nuclear morphology to reflect nuclear changes unrelated to apoptosis, possibly due to altered cell cycle or increased cellular DAPI uptake.

Despite decreasing XIAP embelin treatment did not increase cell death. It is possible that XIAP levels were not sufficiently decreased to disinhibit apoptosis. Alternatively, embelin may have pleiotropic effects on cell death that mask sensitization. Moreover, XIAP may not play a dominant role in apoptosis protection in these cholangiocarcinoma cell lines. This latter explanation seems less likely based on our previous experiments showing that siRNA against XIAP caused increased apoptosis and increased caspase activity in KMCH cholangiocarcinoma cells [6].

Cholangiocarcinoma cell lines exhibited growth inhibition upon treatment with embelin. In Mz-ChA-1 and KMCH cells this was manifested initially as growth arrest at 24 hours. Mz-ChA-1 cells failed to proliferate after this arrest and eventually viability was lost. In KMCH, after the initial 24 hours, the rate of proliferation remained lower than vehicle-treated cells but was not completely halted. HuCCT cells appeared to be resistant to embelin-induced growth arrest. This pattern of strong inhibition in Mz-ChA-1, intermediate inhibition in KMCH, and no effect in HuCCT cells parallels the data on XIAP protein levels. Cell cycle analysis of Mz-ChA-1 cells confirmed an effect of embelin on cell cycle progression, and revealed more cells in S and G2/M phases. This effect is similar to the growth inhibition in PC3 cells where embelin caused a reduction in cells in G0/G1 and increased numbers in S phase and G2/M phase [21]. An increase in the number of cells in the later stages of the cell cycle can be consistent with either increased proliferation, or decreased proliferation due to a late-stage block or slowing in the cell cycle. For instance, cells treated with topoisomerase inhibitor have decreased proliferation and an increased percentage of cells are in both S phase and G2/M (e.g., [35]), consistent with activation of a late checkpoint.

In conclusion, our results demonstrated sensitivity of cholangiocarcinoma cells to treatment with embelin, which resulted in inhibition of cell cycle progression and slowed proliferation. We did not observe increased spontaneous or TRAIL-induced apoptosis in embelin-treated cells, despite reduced XIAP protein levels. In this regard, embelin did cause an alteration in nuclear staining that was initially taken by us to reflect apoptosis. Additional studies on caspase activation as well as cell-by-cell analysis of staining instead revealed altered staining but no increase in characteristic apoptotic nuclear features. Embelin may cause altered cellular uptake of DAPI as untreated healthy cells did not take up this DNA-binding dye. In addition, the effect of embelin to delay cell cycle progression may have resulted in a higher percentage of nuclei in various stages of mitosis manifesting altered nuclear morphology. The late loss of cells that was observed in tetrazolium-based proliferation assays (e.g., Figure 4A at 72 hours) may reflect mitotic collapse, apoptosis, or necrosis. Taken together, our data suggest that embelin may have a growth inhibitory effect in cholangiocarcinoma, but to promote tumor cell apoptosis additional treatments are required.

## Materials and Methods

### Cell Culture and Treatment

Human malignant cholangiocarcinoma cell lines used in this study were KMCH [36], Mz-ChA-1 [37], and HuCCT cells [38]. The highly tumorigenic rat cholangiocarcinoma cell BDEneu was a kind gift from Alphonse Sirica (Virginia Commonwealth University) [39]. Human cells were grown in DMEM with high glucose supplemented with 10% (v/v) fetal bovine serum (FBS), penicillin (100 U/ml), streptomycin (100 µg/ml), G418 (50 µg/ml), and insulin (0.5 µg/ml) at 37°C with 5% CO_2_ in a humidified chamber. BDEneu cells were grown in DMEM supplemented with 10% FBS, human transferrin (5 µg/ml), and insulin (0.5 µg/ml). Embelin was from Sigma-Aldrich and was resuspended in dimethylsulfoxide (DMSO). Staurospirine was from Fisher and was used at 1 µg/mL final concentration. Cells were treated with 0–50 µM embelin for 2–48 hours, as indicated in the figure legends, and compared to DMSO-treated cells (vehicle). Recombinant human TRAIL was obtained from R&D Systems and used at a final concentration of 4–8 ng/mL.

### Immunoblotting

Treated cells were lysed in 50 mM Tris-HCl (pH 7.4), 150 mM sodium chloride, 1 mM ethylenediamine tetraacetic acid, 1 mM dithiothreitol, 1 mM sodium orthovanadate, 100 mM sodium fluoride, and 1% triton X-100 (w/v) supplemented with Complete protease inhibitors. After lysis, insoluble proteins were removed by centrifugation and lysate was separated by sodium dodecylsulfate-polyacrylamide gel electrophoresis (SDS-PAGE), transferred to nitrocellulose, and probed for XIAP or actin. Mouse anti-XIAP antibody (#610717) was from BD Biosciences, and anti-actin antiserum was from SantaCruz. Rabbit anti-PARP antibody (#9542) was from Cell Signaling.

### Cellular thermal shift assay

Mz-ChA-1 cells were grown to 80% confluence and lysed in PBS containing Complete protease inhibitors by three cycles of freeze-thaw (liquid nitrogen), as described [15]. Cell debris was pelleted by centrifugation (13,000 *g* for 20 minutes). Lysates were divided into identical aliquots which were incubated with either embelin (50 µM) or an equal volume of DMSO for 30 minutes and were then heated for 3 minutes on a gradient thermal cycler. The lower temperature was set at 46°C and the higher temperature was at 70°C. Heated samples were then cooled at room temperature for 3 minutes and centrifuged at 13,000 *g* for 20 minutes to pellet denatured protein aggregates. Supernatants were analyzed by SDS-PAGE and immunoblot for XIAP.

### Nuclear Morphology Assay

Treated cells were stained with DAPI (5 µg/mL final) for 20 minutes at 37°C prior to imaging by epifluorescence (Leica DMI6000B). Cells were counted as DAPI-positive if the nucleus showed bright staining, and as apoptotic if there was characteristic nuclear fragmentation, blebbing, or pyknosis. Total cell number was determined in the same field by phase contrast microscopy, and data are expressed as a percent of DAPI-positive nuclei out of total.

### DNA fragmentation assay

Mz-ChA-1 cells were treated with vehicle (DMOS), embelin (15 µM), or staurosporine (1 µg/mL) for 4–24 hours. Fragmented DNA was then isolated essentially following the protocol of Shiraishi et al. [40], except that DNA was extracted by phenol∶chloroform∶isoamyl alcohol prior to RNase A treatment. DNA was run on a 2% agarose gel and visualized by ethidium bromide staining. The image was then digitally inverted and brightness optimized without altering other aspects of the image.

### Caspase 3/7 Assay

Cells were seeded in a 96-well plate and caspase 3/7 activity measured by enzymatic cleavage of a fluorogenic substrate using ApoOne Homogeneous Caspase 3/7 Assay (Promega). The pan-caspase inhibitor Z-VAD-fmk was purchased from Sigma-Aldrich and resuspended in DMSO. Final working concentration was 50 µM.

### Proliferation and Cell Cycle

Cell proliferation was assayed by reduction of 3-(4,5-dimethylthiazol-2-yl)-2,5-diphenyltetrazolium bromide (MTT; Invitrogen). MTT was freshly dissolved into PBS at a stock concentration of 12 mM and diluted into phenol-free DMEM with 10% FBS for a final MTT concentration of 2 mM. Reactions were carried out at 37°C for four hours and stopped by removing the medium. Reduced MTT was dissolved in 100 µL isopropanol and absorbance measured at 540 nm. All data are corrected to the initial signal, set at 100%. Assays were repeated four times for each condition.

### Statistical Analysis

Data were analyzed by ANOVA with post-hoc Bonferroni correction when multiple comparisons were possible. When only two conditions were measured, student's *t*-test was employed. Groups were considered significantly different when the p-value was less than or equal to 0.05.
